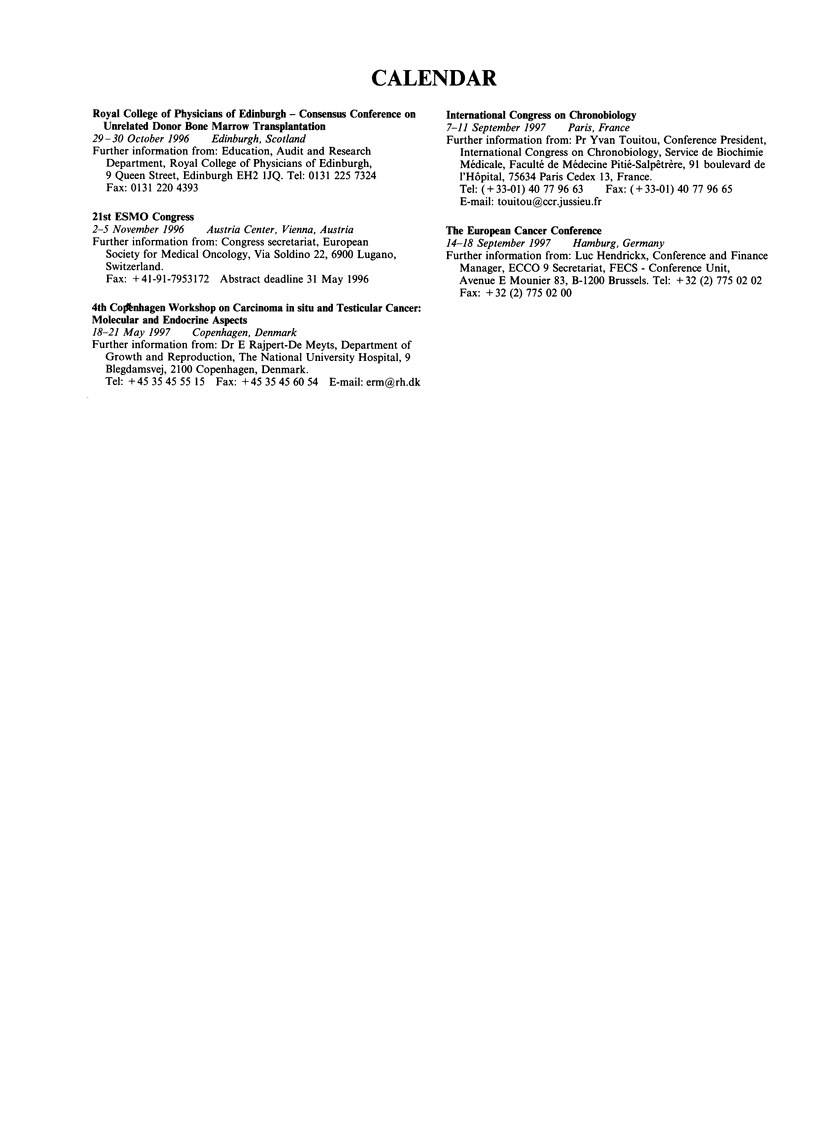# Calendar

**Published:** 1996-11

**Authors:** 


### CALENDAR

**Royal College of Physicians of Edinburgh – Consensus Conference on Unrelated Donor Bone Marrow Transplantation**
*29–30 October 1996 Edinburgh, Scotland*
Further information from: Education, Audit and Research Department, Royal College of Physicians of Edinburgh, 9 Queen Street, Edinburgh EH2 1JQ. Tel: 0131 225 7324 Fax: 0131 220 4393

**21st ESMO Congress**
*2–5 November 1996 Austria Center, Vienna, Austria*
Further information from: Congress secretariat, European Society for Medical Oncology, Via Soldino 22, 6900 Lugano, Switzerland.
Fax: +41-91-7953172 Abstract deadline 31 May 1996

**4th Copenhagen Workshop on Carcinoma in situ and Testicular Cancer: Molecular and Endocrine Aspects**
*18–21 May 1997 Copenhagen, Denmark*
Further information from: Dr E Rajpert-De Meyts, Department of Growth and Reproduction, The National University Hospital, 9 Blegdamsvej, 2100 Copenhagen, Denmark.
Tel: +45 35 45 55 15 Fax: +45 35 45 60 54 E-mail: erm@rh.dk

**International Congress on Chronobiology**
*7–11 September 1997 Paris, France*
Further information from: Pr Yvan Touitou, Conference President, International Congress on Chronobiology, Service de Biochimie Médicale, Faculté de Médecine Pitié-Salpêtrère, 91 boulevard de l'Hôpital, 75634 Paris Cedex 13, France.
Tel: (+33-01) 40 77 96 63 Fax: (+33-01) 40 77 96 65
E-mail: touitou@ccr.jussieu.fr

**The European Cancer Conference**
*14–18 September 1997 Hamburg, Germany*
Further information from: Luc Hendrickx, Conference and Finance Manager, ECCO 9 Secretariat, FECS - Conference Unit, Avenue E Mounier 83, B-1200 Brussels. Tel: +32 (2) 775 02 02 Fax: +32 (2) 775 02 00